# Scabies in Military and Civil Life

**Published:** 1918-09

**Authors:** 


**Scabies in Military and Civil Life ; Its Differentiation, Complications and Treatmeant.** FRANCK CROZER KNOWLES, Captain, M. R. C., U. S. A. Preliminary note of an article to appear elsewhere.

There is probably no greater problem today in the fighting forces than the group of animal parasitic diseases.

During twelve months in a large British General Hospital 1500 of the 2000 dermatological cases were either frank scabies or secondary to the same. The following table will elucidate the differentiation clearly between pediculosis corporis and scabies.

| *Scabies.* | *Pediculosis corporis.* |
|---|---|
| General indistribution, exclusive of the face and scalp. | General indistribution exclusive of the face, scalp, hands, feet, lower arms and the lower legs. |
| Predilection for the webs of fingers. | |
| Diagnostic signs, the burrow, a minute zig-zag line, consisting of alternating blackish and whitish dots. | Diagnostic signs, the pediculus small pin-head in size found in the seams of the clothes more frequently than on the body. |
| The eruption we speak of is multiple, consisting of papules vesicles and pustules, boils and large crusts. | The eruption consists chiefly of long linear scratch marks. Small punctate hemorrhages and excoriations, not infrequently boils and crusts. |
| Itching severe, usually at night. | Itching severe, usually at night. |

In military life scabies is a greater problem than in civil life because of the greater frequency and severity. Hands are involved in but a few cases. The penis usually shows a marked involvement, pustules, and burrows. In civil life complications are absent in most cases, whereas in the army there is an unusually large number of boils, pustules, impetigo and the so-called I. C. T.

*Method of Treating Scabies.*

1st day : Patient is given a warm bath and plenty of soap, one rubbing with sulphur (precipitated sulphur one dram to the ounce of Paraffin molle).

2nd day : Sulphur rubbing.

3rd day : Sulphur rubbing.

4th day : Sulphur rubbing.

5th day : Warm bath with plenty of soap. Clean clothes.

If active lesions are still present four more days of sulphur rubbings are given. A minimum of fifteen minutes is taken for the rubbing of each patient. The ointment is rubbed in with sufficient friction to open up all of the burrows. A thin coating of this ointment is purposely allowed to remain over the entire skin surface, excepting the face and scalp areas.

*Treatment of the Complications of Scabies* :

For all secondary pustular conditions : ammoniated mercury ointment, 20-40 grains to the ounce of paraffin molle. For boils : 25 o/o ichtyol. If boils continue or a large number are present, autogenous vaccines are indicated.

25 o/o of the cases were discharged cured in five days; 50 o/o in nine days; 15 o/o in sixteen days; and the remaining 100/o required three to six weeks.

